# Seasonal hydrology and its impact on buffalo artificial insemination strategies in the amazon region of northern Brazil

**DOI:** 10.1590/1984-3143-AR2025-0062

**Published:** 2026-07-06

**Authors:** Haroldo Francisco Lobato Ribeiro, Sebastião Tavares Rolim, Satish Kumar, Ana Flavia Bezerra da Silva, Luciana Magalhães Melo, Vicente José de Figueirêdo Freitas, William Gomes Vale

**Affiliations:** 1 Setor de Reprodução Animal, Universidade Federal Rural da Amazônia – UFRA, Belém, PA, Brasil; 2 Programa de Pós-graduação em Ciências Veterinárias, Faculdade de Veterinária, Universidade Estadual do Ceará – UECE, Fortaleza, CE, Brasil; 3 Centro de Educação, Ciências e Tecnologia da Região dos Inhamuns, Universidade Estadual do Ceará – UECE, Tauá, CE, Brasil

**Keywords:** Amazon, breeding, buffaloes, FTAI, hydrography

## Abstract

This study aimed to verify how seasonal hydrology affects artificial insemination strategies in northern Brazil to promote better livestock management and ecosystem sustainability. A total of 1780 inseminations were analyzed, including 1590 fixed-time artificial inseminations (FTAI) and 190 conventional artificial inseminations (CAI) (AI in natural estrus without using hormones) conducted over ten years. Of these, 370 inseminations occurred during the flood season (January to May), 700 during the intermediate season (June to September), and 710 during the ebb season (October to January). The estrus synchronization protocols were utilized according the ovarian cyclicity of buffaloes, i.e.: Protocol I (Ovsynch), was used for cycling buffaloes; Protocol II (Ovsynch-P_4_); Protocol III (CL-synch), for the buffaloes with a mature corpus luteum (CL); Protocol IV (P_4_ + BE) + (PGF_2α_ + eCG) + GnRH and Protocol V (P_4_ + VE) + (PGF_2α_ + eCG) + BE were implemented for buffaloes in anestrus, with low body condition, or during unfavorable seasons. AI was performed using cryopreserved-thawed semen. Pregnancy diagnosis was performed using ultrasonography or rectal palpation between 45 and 90 days after insemination. A higher pregnancy rate was observed for the PI (66.87%) than for II (53.77%), III (52.41%), IV (53.35%) and V (33.17%). Regarding seasons, the overall pregnancy rate (53.14%) for FTAI was significantly higher (*P* < 0.05) than CAI (27.4%). During the season of Full tide, Intermediary and Ebb tide, the percentage of pregnancy rate for FTAI vs CAI was 48.28 vs 20.00, 47.77 vs 10.00 and 61.47 vs 41.00, respectively. The pregnancy rate was significantly higher during ebb tide for both FTAI/CAI than in other seasons. In conclusion, seasonal hydrology impacts the outcome of AI strategies in buffaloes in the Amazon region of northern Brazil. A suitable synchronization protocol can enhance buffalo fertility and improve agricultural practices.

## Introduction

The Amazon region of northern Brazil has a complex hydrological system that significantly influences buffalo (*Bubalus bubalis*) husbandry (Sheikh et al., 2006). The hydrography of the Amazon River affects buffalo breeding, with a reproductive season from September to January when conditions are favorable. According to Ribeiro et al. (1999), only 63% of 100 extensively raised buffaloes could be managed for insemination before this period ended, with a pregnancy rate of 59.4%. The geographical challenges, including extensive flooded areas, hinder effective handling during the floods. Consequently, in the Amazon region, only about 0.87% of the herd is inseminated (Ribeiro, 2020).

According to data from the FAO (2022), there are approximately 202 million buffaloes worldwide, with about 97% found in Asian countries such as India, Pakistan, and China. In Brazil, the buffalo population has been increasing steadily over the past few decades, growing remarkably by 127.5% from 1961 to 2000, with an additional 24% increase since then. Today, Brazil boasts the largest buffalo population in the Americas, totaling around 1.4 million animals. Buffalo thrives in the tropical environment and are important economically, but reproductive management presents challenges (Vaz et al., 2024). Artificial insemination (AI) improves reproductive efficiency and genetic diversity, especially when timed with seasonal conditions (Neglia et al., 2020).

Effective reproductive management is essential for enhancing buffalo populations, yet it faces several challenges (Coman et al., 2024). Seasonal rainfall fluctuations affect forage availability and buffalo health. Pasture growth is better during the rainy season, resulting in better nutrition for buffaloes and a higher conception rate. Conversely, the dry season can introduce stress and malnutrition, significantly disrupting fertility and complicating breeding efforts (Vale, 2007). AI has proven to be a significant technique in promoting the genes of elite animals. However, the success of AI depends heavily on applying appropriate estrus synchronization protocols and insemination practices according to the season to ensure successful breeding outcomes (De Rensis and López-Gatius, 2007).

Socio-economic factors further complicate reproductive management, as many farmers lack access to veterinary services (Mumuad, 2013; Işik and Gül, 2016). Education on the significance of seasonal timing in AI and collaboration between researchers and local farmers is vital for improvement. Additionally, seasonal hydrology influences water quality, pathogen levels, and pasture availability, impacting buffalo health (Intawicha et al., 2022). Thus, this study aimed to verify how seasonal hydrology affects AI strategies in northern Brazil to promote better livestock management and ecosystem sustainability.

## Methods

### Ethics

The Ethics Committee on the Use of Animals (# 31032.004688/2024-39) approved this experiment, which follows the guidelines for experimental handling.

### Study period and location

**Figure 1:**
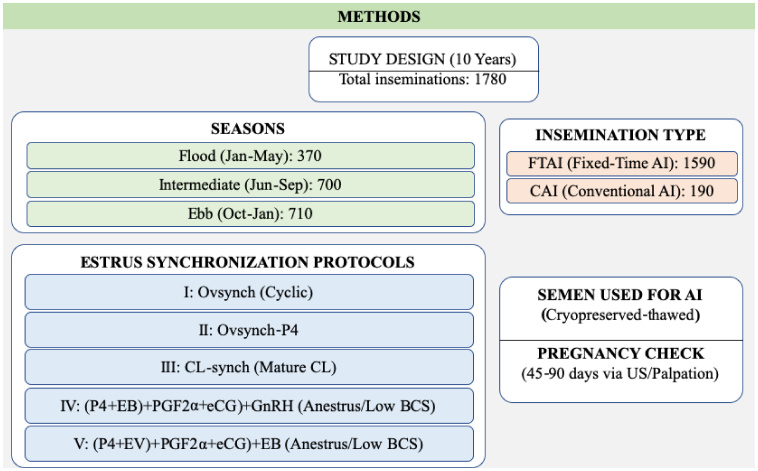
Schematic presentation of the methodology; GnRH: Gonadotropin releasing hormone; PGF_2α_: Prostaglandin F2 alpha; P_4_: Progesterone; EB: Estradiol benzoate; EV: Estradiol valerate; eCG: Equine chorionic gonadotropin; BCS: Body condition score.

**Figure 2:**
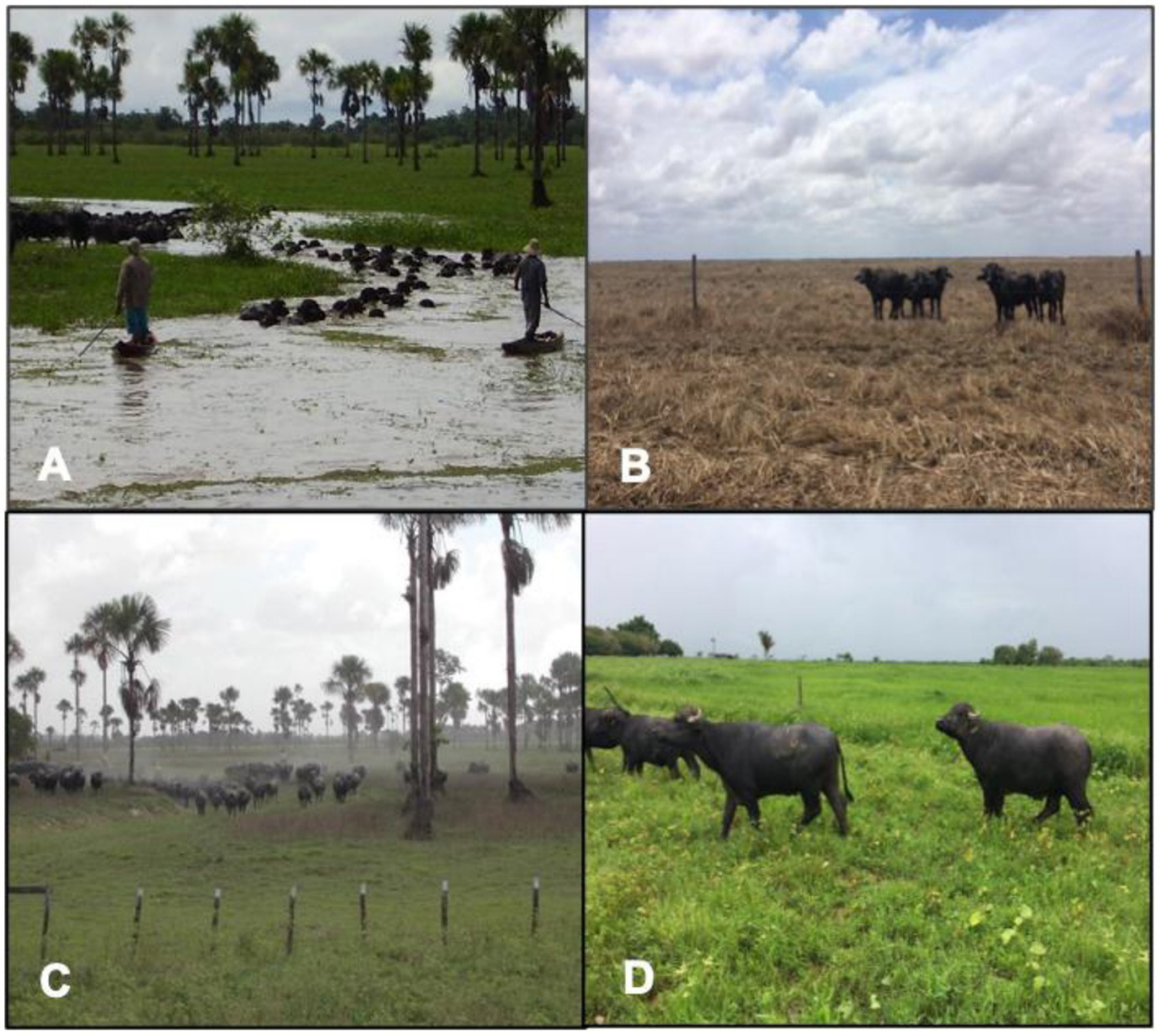
Breeding in the region of the Amazon River and its tributaries during flood season (A), intermediate season (B), and ebb season (C, D).

A total of 1780 inseminations were analyzed, including 190 conventional methods (AI in natural estrus without using hormones) and 1,590 fixed-time artificial inseminations (FTAI) conducted over ten years (Figure 1). Of these, 370 inseminations occurred during the flood season (January to May) (Figure 2A), 700 during the intermediate season (June to September) (Figure 2B), and 710 during the ebb season (October to January) (Figure 2C, D). The body condition score (BCS) of the females ranged from 2 to 4.5 on a scale of 1 to 5 (Singh et al., 2017). This study was conducted at extensive buffalo breeding farms located in Amapá (specifically in the municipalities of Cutias and Itaubal; 0° 59′ N, 50° 48′ W) and Pará state (in the municipalities of Soure, Salvaterra, and Cachoeira do Arari on Marajó Island; 0° 58' S 49° 34' W), Brazil.

### Animal management

The buffaloes were managed in paddocks with native forage (Figure 2). During the ebb season, they fed "Marreco grass" (*Paspalum conjugatum* and *Paspalum virgatum*), while during the flood season, they were fed "Rat tail grass" (*Hymenachne amplexicaulis*) along with "Mururé" (*Eichhornia crassipes*, *Echinochloa repens*, and *Panicum andrequeci*). The females were also fed with adequate mineralized salt. The animals were vaccinated for brucellosis and tuberculosis timely.

### Estrus synchronization protocols, artificial insemination and pregnancy diagnosis

The study utilized several estrus synchronization protocols depending on the ovarian cyclicity of buffaloes (Table 1). Protocol I, known as Ovsynch, was used for cycling buffaloes; Protocol II, Ovsynch-P_4_; Protocol III, CL-synch, for the buffaloes with a mature corpus luteum (CL); and Protocols IV and V were implemented for buffaloes in anestrus, with low body condition, or during unfavorable seasons. Currently, Protocol IV is the most commonly used. AI was done using cryopreserved-thawed semen. Pregnancy diagnosis was performed using ultrasonography or rectal palpation between 45 and 90 days after AI.

**Table 1:** Synchronization protocols used on different farms and their respective application days and doses.

**N^o^**	**D0**	**D3**	**D4**	**D7**	**D8**	**D9**	**D10**	**D11**	**D12**
I	100 µg			500 µg		100 µg	FTAI		
	GnRH			PGF_2α_		GnRH			
II	100 µg			500 µg	P_4_	100 µg	FTAI		
	GnRH +			PGF_2α_	WD	GnRH			
	1.9 g P_4_								
III	500 µg	100 µg	FTAI						
	PGF_2α_	GnRH	(a.m.)						
	(p.m.)	(p.m.)							
IV	1.9 g P_4_ + 2.0 mg EB or 100 µg GnRH					P_4_ WD +500 µg PGF_2α_ + 400IU eCG		100 µg GnRH	FTAI
V	3 mg				P4 WD	1 mg EB	FTAI		
	Norgestomet + 5 mg VE				500 µg PGF_2α_ + 400 IU eCG				

D: Day; GnRH: Gonadotropin releasing hormone; PGF_2α_: Prostaglandin F2 alpha; FTAI: Fixed-time artificial insemination; P_4_: Progesterone; WD: withdraw; EB: Estradiol benzoate; EV: Estradiol valerate; eCG: Equine chorionic gonadotropin; p.m.: Afternoon; a.m.: Morning.

### Data analysis

Data were analyzed using the Chi-square or Fisher's exact test, with a significance level of equal or less than 5%.

## Results

**Figure 3:**
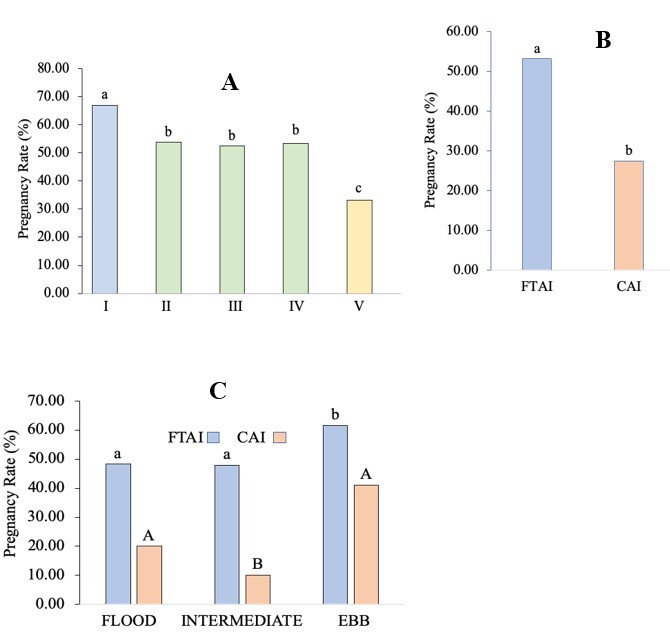
Pregnancy rate according to different protocols (A), overall pregnancy rate between the Fixed-Timed AI (FTAI) and Conventional AI (CAI) (B) and according to different seasons (C) over ten years on extensive breeding farms in the states of Pará and Amapá. Different letters on the bars differ statistically (P < 0.05). Different small and capital letters on the bars of the same season differ statistically (P < 0.05) (C).

A total of 1780 inseminations with five different protocols were analyzed (Table 1, Table 2 and Figure 3A). A higher (*P* < 0.05) pregnancy rate was observed for the Ovsynch protocol (66.87%) than for II (53.77%), III (52.41%), IV (53.35%) and V (33.17%). Regarding seasons, the overall pregnancy rate (53.14%) for FTAI was significantly higher (*P* < 0.05) than CAI (27.4%) (Figure 3B). During the season of Full tide, Intermediary and Ebb tide, the percentage of pregnancy rate for FTAI vs CAI was 48.28 vs 20.00; 47.77 vs 10.00 and 61.47 vs 41.00, respectively (Table 3 and Figure 3C). The pregnancy rate was significantly higher during Ebb tide for both FTAI / CAI than in other seasons.

**Table 2:** Pregnancy rate according to different protocols, over a ten-year period on extensive breeding farms in the states of Pará and Amapá.

**No.**	**Protocols**	**Pregnancy rate (%)**
I	Ovsynch	66.87^a^ (210/314)
II	Ovsynch + P_4_	53.77^b^ (185/344)
III	CL-Synch	52.4^b^ (195/372)
IV	(P_4_ + BE) + (PGF_2α_ + eCG) + GnRH	53.35^b^ (183/343)
V	(P_4_ + VE) (PGF_2α_ + eCG) + BE	33.17^c^ (72/217)
	Total	53.14 (845/1590)

Different letters in the same column differ statistically (*P* < 0.05).

**Table 3:** Pregnancy rate according to hydrography of the Amazon valley, over a ten-year period on extensive management farms in the states of Pará and Amapá.

**Seasons**	**Pregnancy rate (%)**	
	**FTAI**	**Conventional AI**
Full tide (February-May)	48.28 (169/350)	20.00 (4/20)
Intermediary (June-September)	47.77 (301/630)	10.00 (7/70)
Ebb tide (October-January)	61.47 (375/610)	41.00 (41/100)
Total	53.14^a^ (845/1590)	27.4^b^ (52/190)

FTAI: Fixed-time artificial insemination. Different letters in the same row differ statistically (*P* < 0.05).

## Discussion

In the present study we highlighted the seasonal hydrological effect on efficiency of AI in buffaloes and managemental strategies to improve the AI results.

The effectiveness of the protocol I (Ovsynch) was represented by the pregnancy rate of 66.87% (Table 2) in cyclic buffaloes. Couto (2007) supported our results and obtained a 66.67% pregnancy rate using Ovsynch, in the same geographical condition of our study, assessing buffaloes with a BCS of 3.0 to 3.5. In contrast, Picanço (2006) and Baruselli and Carvalho (2005) reported 60 and 55.4% of pregnancy rates, respectively, in extensive breeding during low-water seasons, which was slightly lower than our results. Studies stated that the BCS influenced the outcome of estrus synchronization protocols (Couto, 2007; Baruselli et al., 2009).

In our study, it was observed a 53.77% pregnancy rate using protocols combining intravaginal progesterone/progestins with Ovsynch. Treatments of progesterone/progestins with EB, eCG, and GnRH facilitate FTAI during unfavorable reproductive seasons, decreasing the anestrus period in buffaloes (Neglia et al., 2003). Moreover, Rhodes et al. (2002) demonstrated that progesterone treatment in anestrus cows enhances estradiol production by dominant follicles, increasing LH pulsatile release. This treatment raises the number of LH receptors in granulosa and theca cells of preovulatory follicles, fostering follicular growth and ovulation, thereby enhancing reproductive efficiency in anestrus females. Ribeiro et al. (2003) achieved a higher pregnancy rate (65.5%) and Vale et al. (2006) reported 55.56%. Wiltbank et al. (2011) noted that high circulating progesterone levels before FTAI are essential for maximizing dairy cow fertility. Colazo et al. (2013) found that progestin supplementation with Ovsynch enhances pregnancy rate compared to classic Ovsynch, especially in the presence of non-cyclic cows. Wiltbank et al. (2010) suggested that elevated progestin levels during follicular development improve fertility by reducing codominant follicle selection and double ovulation.

It was obtained a 52.41% pregnancy rate using the CL-synch protocol. This protocol was used in cyclic, good BCS and without a calf at their feet. Sousa et al. (1999) obtained 75% of the pregnancy rate using prostaglandin and GnRH in buffaloes without a calf at their feet, BCS ≥ 3.0 and cycling. In another study, Nunes et al. (2010) obtained a 42.79% pregnancy rate using 150µg D-cloprostenol (D0), 100µg GnRH seventy-two hours later (D3) and FTAI simultaneously.

In this study, we obtained a 53.35 and 33.17% pregnancy rate using protocols IV and V, respectively. The significantly lowest pregnancy rate (33.17%) was obtained using a protocol associated with EV (Table 2). There has already been a description that EV inhibits follicular growth and ovulation in bubaline (Bartolomeu et al., 1999).

Regarding seasons, the overall pregnancy rate (53.14%) for FTAI was higher significantly (*P* < 0.05) than CAI (27.4%) (Table 3). During the season of Full, Intermediary and Ebb tide, the percentage of pregnancy rate for FTAI vs CAI was 48.28 vs 20.00, 47.77 vs 10.00 and 61.47 vs 41.00, respectively (Table 3). The pregnancy rate was significantly higher during Ebb tide for FTAI / CAI than in other seasons. These results indicated that the hydrographic area influenced (*P* < 0.05) pregnancy rate. The overall lower pregnancy rate during the seasons of Full tide or Intermediary for CAI is due to difficulties in estrus detection, insemination logistics and management of buffaloes during these seasons. Ribeiro et al. (1999) worked with 100 buffaloes in flood-prone areas and were able to manage and inseminate only 63. Out of these females, the authors only successfully diagnosed 50%, where 59.4% were pregnant. Moreover, the low percentage of insemination and diagnosis of buffaloes is due to the geographical location of the area, which includes flooded riverbank areas, making it impossible to handle them properly at certain times, i.e. during the beginning and end of floods) (Ribeiro, 2002; Picanço, 2006).

Different studies observed that the hydrography of the Amazon River and its tributaries determine that regional buffalo breeding a limited period of reproduction, varying from three to five months, between October and January, a period of the ebb of the rivers, time of easy handling and abundant availability of native pastures, representing the favorable season for reproduction. On the other hand, the intermediate seasons (Jun to September) and the flood season (February to May) are limiting the practice of AI (Ribeiro, 2002; Ribeiro et al., 2003).

Aprile and Darwich (2013) reported that during floods, the rivers of the Amazon region invade marginal areas. They also noted that appreciable quantities of mineral and organic sediments in their waters are deposited on the floodplains, giving them great fertility and value for intensive food production. In the flood season, extensive farmlands in the Amazon are flooded, and pastures are submerged, destroying terrestrial feed and making aquatic feed available (Kroth et al., 2015).

In the other parts of Amazon, Ribeiro et al. (2003) and Vale et al. (2006) support our results. They obtained 57.5% and 49.8% of pregnancy rates during the favorable and unfavorable periods, respectively, after FTAI. Moreover, Couto (2007) observed 58.82% and 49.15% of pregnancy rates during the rainy and the least rainy periods, respectively, after FTAI. Thermal stress influences reproductive performance in the less rainy season in this area (Silva et al., 2014). During favorable seasons, animals are more comfortable, which leads to more satisfactory follicular development and increased pregnancy rates after FTAI (Garcia, 2013).

## Conclusion

Seasonal hydrology impacts the outcome of AI strategies in buffaloes in the Amazon region of northern Brazil. However, a suitable synchronization protocol can enhance buffalo fertility and, consequently, improve agricultural practices.

## Data Availability

Research data is only available upon request.
